# Development and validation of a nomogram model for mortality prediction in stable chronic obstructive pulmonary disease patients: A prospective observational study in the RealDTC cohort

**DOI:** 10.7189/jogh.14.04049

**Published:** 2024-02-23

**Authors:** Wei Cheng, Aiyuan Zhou, Qing Song, Yuqin Zeng, Ling Lin, Cong Liu, Jingcheng Shi, Zijing Zhou, Yating Peng, Jing Li, DingDing Deng, Min Yang, Lizhen Yang, Yan Chen, Shan Cai, Ping Chen

**Affiliations:** 1 Department of Pulmonary and Critical Care Medicine, Second Xiangya Hospital; Research Unit of Respiratory Disease; Diagnosis and Treatment Centre of Respiratory Disease, Central South University, Changsha, Hunan, China; 2 Clinical Medical Research Centre for Respiratory and Critical Care Medicine in Hunan Province, China; 3 Department of Pulmonary and Critical Care Medicine, Xiangya Hospital, Central South University, Changsha, Hunan, China; 4 Department of Epidemiology and Health Statistics, Xiangya School of Public Health, Central South University, Changsha, China; 5 Department of Respiratory Medicine, The First Affiliated People's Hospital, Shaoyang College, Shaoyang, Hunan, China

## Abstract

**Background:**

Chronic obstructive pulmonary disease (COPD) is the third leading cause of death worldwide. There is no nomogram model available for mortality prediction of stable COPD. We intended to develop and validate a nomogram model to predict mortality risk in stable COPD patients for personalised prognostic assessment.

**Methods:**

A prospective observational study was made of COPD outpatients registered in the RealDTC study between December 2016 and December 2019. Patients were randomly assigned to the training cohort and validation cohort in a ratio of 7:3. We used Lasso regression to screen predicted variables. Further, we evaluated the prognostic performance using the area under the time-dependent receiver operating characteristic curve (AUC) and calibration curve. We used the AUC, concordance index, and decision curve analysis to evaluate the net benefits and utility of the nomogram compared with three earlier prediction models.

**Results:**

Of 2499 patients, the median follow-up was 38 months. The characteristics of the patients between the training cohort (n = 1743) and the validation cohort (n = 756) were similar. ABEODS nomogram model, combining age, body mass index, educational level, airflow obstruction, dyspnoea, and severe exacerbation in the first year, was constructed to predict mortality in stable COPD patients. In the integrative analysis of training and validation cohorts of the nomogram model, the three-year mortality prediction achieved AUC = 0.84; 95% confidence interval (CI) = 0.81, 0.88 and AUC = 0.80; 95% CI = 0.74, 0.86, respectively. The ABEODS nomogram model preserved excellent calibration in both the training cohort and validation cohort. The time-dependent AUC, concordance index, and net benefit of the nomogram model were higher than those of BODEx, updated ADO, and DOSE, respectively.

**Conclusions:**

We developed and validated a prognostic nomogram model that accurately predicts mortality across the COPD severity spectrum. The proposed ABEODS nomogram model performed better than earlier models, including BODEx, updated ADO, and DOSE in Chinese patients with COPD.

**Registration:**

ChiCTR-POC-17010431.

Chronic obstructive pulmonary disease (COPD) is a chronic respiratory disease resulting in respiratory symptoms and airway obstruction [1]. COPD is already the third leading cause of death worldwide, causing 3.23 million deaths in 2019 [2,3]. COPD is a rather heterogeneous disease, and stratifying patients by prognosis will improve the likelihood of a precision medicine approach.

Previous studies have reported that increased age is associated with a poor prognosis in COPD, with age over 68 years being one of the predictors of future deterioration risk [4,5]. A review reported that consistently strong predictors were forced expiratory volume in one second percentage predicted (FEV_1_ percentage predicted) and age in stable COPD [6]. Low levels of body mass index (BMI), body weight reduction, lower FEV_1_ at baseline, and high symptom scores are associated with a higher risk of COPD mortality [7-9]. Previous severe acute exacerbation leads to an increased risk of hospitalisation and mortality in COPD patients [10]. Previous studies found that higher education was related to lower mortality [11,12]. The above-mentioned single predictors or risk factors provide limited predictive value for disease prognosis because several clinical phenotypes exist in COPD patients [1]. Composite models or indexes have the potential to explain many aspects of COPD [13]. Global Strategy for the Diagnosis, Management, and Prevention of Chronic Obstructive Lung Disease (GOLD) recommends using multivariable prediction models to assess the prognostic profile and facilitate follow-up of patients instead of single predictors such as the above predictors [1,14].

BODE index is a multidimensional index constructed with BMI, airflow obstruction, dyspnoea, and exercise capacity [15]. The previous study exhibited that the BODE index had a similar prognostic capacity to the BODEx index including exercise capacity [16]. Due to the large number of outpatients in China, the feasibility of the exercise capacity experiment, such as the six-minute walking distance, is low. Therefore, we chose to compare BODEx instead of BODE to the nomogram model [16]. Updated ADO index combines age, dyspnoea, and airflow obstruction to predict the risk of mortality across all the COPD severity [17]. DOSE index includes dyspnoea, airflow obstruction, current smoking status, and exacerbation frequency in the past year, which was associated with mortality in COPD patients [18,19]. These indexes are three other major risk assessment systems. A nomogram prognostic model for predicting patient prognosis may contribute to better treatment stratification and clinical outcome assessment [20]. Many prognostic models combining multiple predictors have been developed for outcome prediction in COPD patients, but more than 80% of prognostic models were not calibrated [14]. China is a developing country with lower education levels in the elderly, low cognitive levels, and low awareness of symptoms in COPD patients [21]. Smoking and biomass fuels are the main risk factors for COPD in China [22]. The previous study also showed that the proportion of COPD patients with high acute exacerbation risk and high symptoms is high in China [23]. However, no such nomogram model is available for mortality prediction of stable COPD.

Hence, we intended to identify prognostic factors for COPD patients in China and then develop and validate a new nomogram prognostic model to predict mortality risk in stable COPD patients. We also compared the predictive value of the ABEODS nomogram model with three earlier prediction models (BODEx, the updated ADO, and DOSE).

## METHODS

### Study participants

This is a prospective observational study named RealDTC study (the analysis of current status in diagnosis and treatment of COPD), which has been reported previously [24]. Participants were included between December 2016 and December 2019 and randomly assigned to the training cohort and validation cohort in a ratio of 7:3. COPD was defined with the FEV_1_ divided by the forced vital capacity (FVC) after bronchodilation less than 0.70 according to the GOLD 2017 recommendations [1]. Patients at least 40 years old were included. Patients previously diagnosed with asthma, bronchiectasis, interstitial lung disease, carcinoma, severe heart diseases, chronic liver diseases, or chronic kidney diseases were excluded (based on personal medical records). Patients who had an acute exacerbation (AE) of COPD within one month before the visit to the outpatient clinic were also excluded. This study was approved by the local ethics committee of the Second Xiangya Hospital (number 2016076). Informed consent was obtained from all participants.

### Variables and definitions

We collected at baseline age, sex, BMI, smoking status, pulmonary function tests, COPD assessment test (CAT) score, modified medical research council dyspnoea scale (mMRC) score, clinical COPD questionnaire (CCQ) score, number of AE in the past year, the severity of exacerbation (moderate or severe) and inhaled drug prescription. Smoker was defined as a person with a current or past smoking history of more than 10 packs per year. Patients who had sustained abstinence for more than six months were former smokers [25]. Never-smokers were defined as those who had a lifetime exposure to no more than 100 cigarettes or no more than 10 packs per year [26]. The post-bronchodilator FEV_1_ is a percent of the predicted value, classified according to the stages identified by the American Thoracic Society because it can be used to predict health status [27], and the risk of death [8]. This classification (≤35, 36–50, 51–64, ≥65) of FEV1 percentage predicted was applied to the BODE index [15]. We defined moderate exacerbation as requiring a prescription for an oral corticosteroid and/or antibiotic. Severe exacerbation requires hospital management, including emergency visits or admissions [1]. The AE frequency and severity in the past year were obtained retrospectively, but the first year AE frequency and severity were obtained prospectively. We defined ABEODS as a combination of age, BMI, educational level, airflow obstruction, dyspnoea, and severe exacerbation frequency in the first year. BODE index is a multidimensional 10-point scale constructed with BMI, airflow obstruction, dyspnoea, and exercise capacity [15]. BODEx combines BMI, airflow obstruction, dyspnoea, and severe exacerbation frequency in the past year, which has a similar prognostic capacity to the BODE index [16]. The score range for this index is between zero and nine points [16]. The updated ADO index, ranging from zero to 14 points, combines age, dyspnoea, and airflow obstruction to predict mortality risk across all the COPD severity [17]. DOSE index includes four components – dyspnoea, airflow obstruction, current smoking status, and exacerbation frequency in the past year – which was associated with mortality in COPD patients [18,19].

We followed up patients every six months after the first visit to the outpatient clinic. The number of exacerbations, the severity of exacerbation (moderate or severe), and survival status were collected per follow-up. We defined all-cause mortality as the outcome. Further, we defined overall survival (OS) as the length of time from the first visit to the outpatient clinic to death or last contact. These were obtained from personal follow-up. The study endpoint was death or the last routine follow-up before 31 October 2022.

### Sample size estimation

We calculated the sample size using PASS, version 15.0 (Kaysville, Utah, USA) in the part of Cox Regression. We used the mortality (7.80%), R-squared as 0.074, standard deviation as 0.188 obtained from the pre-experiment, set the interval type as two-sided, and entered the confidence level (1–Alpha) as 0.80 and confidence interval (CI) width (two-sided) as 0.05. Finally, the sample we acquired was 1134.

### Statistical analysis

We drew a normal quantile-quantile plot to test whether the distribution of the quantitative data follows the normal distribution. Data meeting the normal distribution were presented as mean and standard deviation (SD), otherwise as median and interquartile range. For normally distributed data, we used a *t* test for comparison between the two groups. Otherwise, we used the Mann-Whitney U-test. Categorical variables were expressed by frequency. We used the χ^2^ test to compare the categorical variables of different groups and Lasso regression to screen predicted variables for inclusion in the nomogram. Moreover, we evaluated the OS probabilities by nomogram. We used the concordance index (C-index) and/or area under the time-dependent receiver operating characteristic (ROC) curve (time-dependent AUC) to evaluate discriminative ability. Further, we used calibration curves to evaluate calibrating ability. The cut-off value of variables was based on Youden’s index from ROC curve analysis. C-index and/or area under the ROC curve (AUC) more than 0.7 suggest a rational estimation. We used the changes in the net reclassification index and integrated discrimination improvement to compare the accuracy between the nomogram and other prediction models, including BODEx, updated ADO, and DOSE. Time-dependent AUC, C-index, and decision curve analysis were used to evaluate the net benefits and utility of the nomogram model compared with other prediction models. We employed the Kaplan-Meier and multivariable Cox regression analyses for survival analysis. We used a method developed by Hanley and McNeil to compare ROC curves derived from the same subjects [28]. We performed all analyses with the statistical software *R*, version 4.1.2 (R Core Team, Vienna, Austria), Free Statistics, version 1.7.1 (Freeclinical Ltd, Beijing, China), and MedCalc, version 19.7.2 (MedCalc Software Ltd, Ostend, Belgium). A *P*-value <0.05 was considered statistically significant.

## RESULTS

### Subject characteristics

2874 stable COPD patients were included at baseline, and 2499 patients were included for analysis (375 refused to follow up). The median follow-up periods were 38.0 (36.0–46.0) months. The characteristics of the patients between the training cohort (n = 1743) and the validation cohort (n = 756) were similar (Table S1 in the **Online Supplementary Document**).

Of 1743 patients in the training cohort, 1529 (87.7%) were male patients, with a mean age of 63.1 (SD = 8.0) and a mean FEV_1_ percentage predicted of 53.0 (SD = 21.0%). A total of 116 (6.7%) deaths were recorded. There were significant differences in age, BMI, educational level, CAT, mMRC, CCQ, FEV_1_/FVC, FEV_1_, FEV_1_ percentage predicted, COPD severity, GOLD 2017 ABCD group classification, AE/severe AE frequency in the past year, AE/severe AE frequency in first year follow up between survivors and non-survivors (Table S2 in the **Online Supplementary Document**). Besides, the indexes of BODEx, updated ADO, and DOSE were lower among survivors than non-survivors.

### Nomogram variable screening, construction, and validation

We included the above baseline differential indicators, sex, and smoking status for Lasso regression screening (**Table 1**). Furthermore, the comparison of ROC curve analysis demonstrated that the predictive value of AE during the first year for COPD mortality was higher than that for AE (*P* = 0.02) and severe AE in the past year (*P* < 0.01). The predictive value of severe AE in the first year for COPD mortality was higher than for AE in the first year (*P* < 0.01). (Figure S1 in the **Online Supplementary Document**).

**Table 1:** Screening predicted variables based on Lasso regression in COPD training cohort*

Variable*	HR (95% CI)	*P*-value
Age in years	1.06 (1.04, 1.09)	<0.01
BMI	0.91 (0.86, 0.96)	0.01
Educational level		
*Primary/below*	ref	
*Middle*	0.57 (0.37, 0.90)	0.01
*High*	0.57 (0.31, 0.99)	0.05
*College/upper*	0.23 (0.06, 1.01)	0.04
FEV_1_ percentage predicted		
*>46.3*	ref	
*≤46.3*	1.65 (0.84, 2.79)	0.02
mMRC	1.45 (1.06, 1.83)	0.01
Severe AE frequency in the first year	1.42 (1.24, 1.88)	<0.01

AE – acute exacerbation, BMI – body mass index, CI – confidence interval, FEV_1_ – forced expiratory volume in one second, HR – hazard ratio, mMRC – modified medical research council dyspnoea scale, ref – reference

*COPD severity and group classification were classified using Global Initiative for Chronic Obstructive Lung Disease criteria.

**Figure 1:**
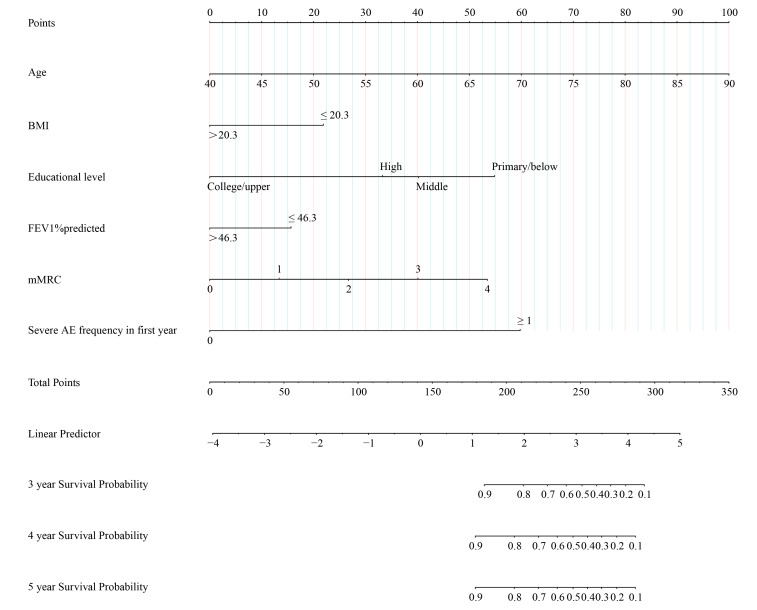
ABEODS nomogram model to predict the risk of mortality in stable COPD. ABEODS – combination of age, BMI, educational level, airflow obstruction, dyspnoea and severe exacerbation in the first year, BMI – body mass index, COPD – chronic obstructive pulmonary disease, FEV_1_ – forced expiratory volume in one second, mMRC – modified medical research council dyspnoea scale.



The cut-off value of FEV_1_ percentage predicted was 46.3% based on Youden’s index from the ROC curve (AUC = 0.62; 95% CI = 0.59, 0.64, *P* < 0.01). The cut-off value of BMI was 20.3 based on Youden’s index from the ROC curve (AUC = 0.64; 95% CI = 0.61, 0.66, *P* < 0.01). The cut-off value of severe exacerbation frequency was one based on Youden’s index from the ROC curve (AUC = 0.71; 95% CI = 0.68, 0.73, *P* < 0.01). In Cox regression analysis, the hazard ratios (HR) for mortality of variables including age, BMI, educational level, degree of airflow obstruction, dyspnoea, and severe exacerbation are shown in **Table 2**. The ABEODS nomogram model was constructed to predict the risk of three, four, and five-year mortality in stable COPD according to these independent risk factors (**Figure 1**). We calculated the total points for individuals’ scores by using this nomogram. We obtained the corresponding survival probability by the corresponding points in the nomogram. The formula for calculating the survival probability from the nomogram was:

**Table 2:** Hazard ratios for mortality of variables used in the ABEODS nomogram model in the training cohort

Item	HR (95% CI)	*P*-value
Age in years	1.06 (1.03, 1.09)	<0.01
BMI		
*>20.3*	ref	
*≤20.3*	1.87 (1.29, 2.71)	0.01
Educational level		
*Primary/below*	ref	
*Middle*	0.66 (0.43, 1.01)	0.06
*High*	0.54 (0.30, 0.96)	0.04
*College/upper*	0.21 (0.05, 0.86)	0.03
FEV_1_ percentage predicted		
*>46.3*	ref	
*≤46.3*	1.56 (1.04, 2.36)	0.03
mMRC	1.47 (1.17, 1.83)	0.01
Severe AE frequency in the first year		
*0*	ref	
*≥1*	5.52 (3.78, 8.07)	<0.01

AE – acute exacerbation, BMI – body mass index, CI – confidence interval, FEV_1_ – forced expiratory volume in one second, HR – hazard ratio, mMRC – modified medical research council, ref – reference

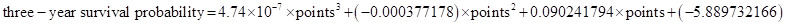





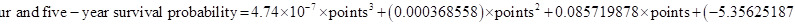



The AUC predicting three, four, and five-year survival likelihood was 0.84 (95% CI = 0.81, 0.88), 0.83 (95% CI = 0.76, 0.90), and 0.86 (95% CI = 0.77, 0.95), respectively in the training cohort. As demonstrated in the validation cohort, the AUC was more than 0.75 for predicting three-year and four-year survival probability. However, the discriminate ability in predicting five-year survival probability was poor (AUC = 0.59). The calibration curves of three, four, and five-year OS probability showed high consistency between predicted survival probability and observed survival probability in these two cohorts (Figure S2–3 in the **Online Supplementary Document**).

### Clinical value of the nomogram model compared with three earlier prediction models

The changes in the net reclassification index were more than zero between the nomogram model and BODEx. The changes in the net reclassification index and integrated discrimination improvement were more than zero between the nomogram model and the updated ADO model. A similar trend was also found in the comparison of the nomogram model and DOSE (**Table 3**). The time-dependent AUC and C-index of the nomogram model were higher than those of the BODEx, updated ADO, and DOSE, respectively. Decision curve analysis showed that the nomogram model could better predict the three, four, and five-year survival probability, as it added more net benefits compared with these three earlier prediction models for almost all threshold probabilities (Figure S4–5 in the **Online Supplementary Document**).

**Table 3:** Net reclassification index and integrated discrimination improvement analysis between nomogram and three existing models in mortality prediction in the training cohort

	Estimate (95% CI)	*P*-value
**ABEODS nomogram model vs BODEx model**		
Integrated discrimination improvement	0.03 (-0.01, 0.06)	0.07
Net reclassification index	0.28 (0.13, 0.39)	<0.01
Median improvement in risk score	0.04 (0.01, 0.06)	<0.01
**ABEODS nomogram model vs updated ADO model**		
Integrated discrimination improvement	0.02 (0.01, 0.04)	<0.01
Net reclassification index	0.26 (0.08, 0.36)	<0.01
Median improvement in risk score	0.02 (0.01, 0.04)	<0.01
**ABEODS nomogram model vs DOSE model**		
Integrated discrimination improvement	0.03 (0.01, 0.05)	0.01
Net reclassification index	0.31 (0.15, 0.41)	<0.01
Median improvement in risk score	0.03 (0.01, 0.04)	<0.01

ABEODS – a combination of age, BMI, educational level, airflow obstruction, dyspnoea, and severe exacerbation in the first year, updated ADO – a combination of age, dyspnoea, obstruction, BMI – body mass index, BODEx – a combination of BMI, airflow obstruction, dyspnoea, and exacerbations, CI – confidence interval, DOSE – a combination of the dyspnoea, obstruction, smoking, exacerbation

### Construction and Cox regression analysis of the ABEODS index

To apply the ABEODS model more conveniently to clinical practice, we scored the metrics of this model quantitatively (**Table 4**). The cut-off value of age was 62 based on Youden’s index from ROC curve analysis (AUC = 0.68; 95% CI = 0.65, 0.70, *P* < 0.01). The value for mMRC was consulted for the categories used in BODE. We estimated these values based on the points in the nomogram including age, BMI, educational level, FEV_1_ percentage predicted, mMRC, and severe exacerbation frequency. The score range for this index was between zero and 14 points. The following quartiles were considered: quartile one (0–5 points), quartile two (6–7 points), quartile three (8–9 points), and quartile four (≥10 points).

**Table 4:** Variables and point values used for the computation of the ABEODS index

	Points on ABEODS index*			
**Variables**	**0**	**1**	**2**	**3**
Age in years	40–62			≥63
BMI	≥20.3	<20.3		
Education level	College/upper	High	Middle	Primary/below
FEV_1%_ predicted	>46.3	≤46.3		
mMRC	0–1	2	3	4
Severe exacerbation in the first year	0			≥1

ABEODS – a combination of age, BMI, educational level, airflow obstruction, dyspnoea and severe exacerbation in the first year, BMI – body mass index, FEV_1_ – forced expiratory volume in one second, mMRC – modified medical research council dyspnoea scale

*The score range for this index is between zero and 14 points. The cut-off values of age, BMI, FEV_1_ percentage predicted, and severe exacerbation are based on Youden’s index from the receiver operating characteristic curve. The education level categories are based on the Cox regression model. The value for mMRC was consulted for the categories used in BODE.

**Figure 2:**
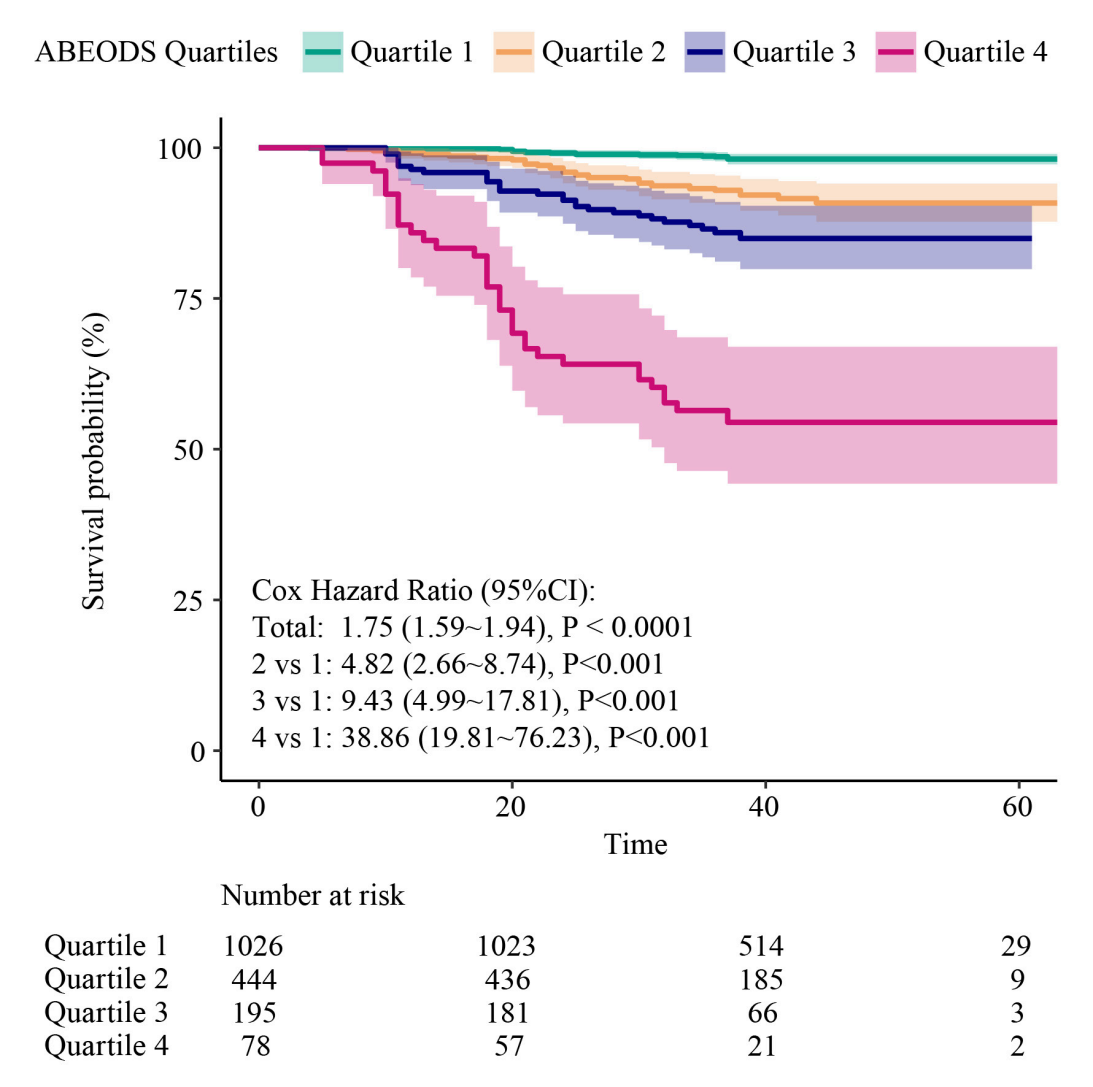
Kaplan-Meier overall survival curves for the four quartiles of the ABEODS index. The score range for this index is between zero and 14 points. The following quartiles were considered: quartile one (0–5 points), quartile two (6–7 points), quartile three (8–9 points), and quartile four (≥10 points). ABEODS – a combination of age, BMI, educational level, airflow obstruction, dyspnoea, and severe exacerbation in the first year, BMI – body mass index, CI – confidence interval.

To apply the model to clinical practice, we scored and stratified the indicators in the model. Patients with higher ABEODS scores were at higher risk for death; the HR for death from any cause per one-point increase in the ABEODS score was 1.75. We also compared the strata by using the Kaplan-Meier analysis. The adjusted mortality risk increased 4.82 times for patients classified to quartile two when compared with patients classified to quartile one. The adjusted mortality risk increased 9.43 times for patients classified to quartile three when compared with patients classified to quartile one. The adjusted mortality risk increased 38.86 times for patients classified to quartile four when compared with patients classified to quartile one. Finally, we defined patients in quartile one as low risk, patients in quartiles two and three as moderate risk, and patients in quartile four as high risk. (**Figure 2**). Further survival analysis adjusted for different variables at baseline found that patients classified as moderate risk (HR = 6.00; 95% CI = 3.43, 10.47, *P* < 0.01) and high risk (HR = 36.89; 95% CI = 18.82, 72.33, *P* < 0.01) in ABEODS had a significantly higher mortality risk than patients classified to low risk.

### Comparison among the ABEODS index and three earlier indexes

The AUC of the ABEODS index was higher than the AUC of other composite predictor indexes, such as the BODEx (*P* < 0.01), the updated ADO (*P* < 0.01), and the DOSE (*P* < 0.01) (Figure S6 in the **Online Supplementary Document**).

## DISCUSSION

In this study, we aimed to develop and validate a prognostic nomogram model for mortality prediction in stable COPD patients. This nomogram model can be used to predict mortality in COPD individuals, achieving individualised prognostic assessment and providing some value for adjusting treatment assessment.

Predictor variables were screened based on the Lasso regression, and the nomogram model ultimately included severe AE frequency during first-year follow-up rather than AE frequency in the past year. The data on AE frequency in the past year was recalled by the patient, and it may be inaccurate [29], which will reduce the predictive value of mortality. Furthermore, the predictive value of severe AE in the first year for mortality was higher than that for AE in the first year. A 17-year follow-up cohort study exhibited that every new severe exacerbation increases the risk of death, the peak does not rise much more after the third severe exacerbation [10]. Juan et. al found that severe exacerbation during the first year follow-up presented as an independent adverse prognostic factor [16]. These studies further provide an explanation for the better predictive value of severe exacerbation frequency over the number of exacerbations during the first year.

In the Cox prediction regression model, the risk of death among patients with primary or below educational level was more than four times higher than that at college or above. Unlike other common prediction models, our nomogram model incorporates educational levels into the prediction model. Previous studies demonstrated that a higher educational level was associated with the mortality of COPD [11,12]. Educational level is one of the indexes of socioeconomic status [30] which may affect the patient’s ability to bear the disease burden. Study showed that socioeconomic status affects the risk of health conditions including multimorbidity, frailty, and disability [30]. Bartosz et. al found that a lower educational level was an independent predictor of poor therapy adherence [31]. Poor medication adherence to inhaled corticosteroids and tiotropium was associated with a higher risk of mortality [32]. These findings further support the association of educational level with COPD mortality.

Patients who deceased had higher CAT, CCQ, and mMRC scores than survivors. ROC analysis presented that higher CAT (AUC = 0.65; 95% CI = 0.62, 0.67), CCQ (AUC = 0.63; 95% CI = 0.61, 0.65), and mMRC (AUC = 0.67; 95% CI = 0.65, 0.69) scores were related to higher mortality. The above results are consistent with those of the previous study [9,33]. Lasso regression selected mMRC as one of the variables of the nomogram prediction model, which is consistent with the composition of other earlier models such as BODE, BODEx, ADO, updated ADO, and DOSE [14–18]. There was no difference between the baseline smoking status of the dead COPD patients and the survivors, and this indicator was ultimately not included in the prediction model. As we know, smoking cessation is an effective intervention in increasing survival and reducing morbidity [34]. In the real world, there is uncertainty about smoking cessation during follow-up, which we believe may be one of the reasons why the baseline smoking status is not included in the prediction model.

According to the time-dependent ROC and DCA analysis, the proposed ABEODS nomogram model performed better than earlier models, including BODEx, updated ADO, and DOSE in COPD patients. In addition to these existing prediction models, a previous study conducted on a machine-learning mortality prediction model outperformed four existing models for predicting all-cause mortality across two COPD cohorts [35]. However, the indicators required by the model are numerous; some indicators, such as six-minute walking distance and oxygen saturation, are difficult to obtain in the outpatient department routine, which may reduce the clinical application value of the model. Other machine-learning models for the prediction of mortality in COPD included imaging parameters [36–38]. Although such prediction models have good prediction ability for COPD patients, patients must finish the chest computed tomography scanning, and imaging professionals to evaluate the relevant impact indicators of emphysema to complete the prediction of mortality. The above factors may lead to the model being less practical and convenient in clinical application than our model. Moreover, the predictive value of the ABEODS index was higher than that of the BODEx index, updated ADO index, and DOSE index which further proved that the ABEODS index has a good predictive value for COPD mortality.

The validation cohort exhibited that the predictive ability was strong for predicting the three-year and four-year survival probability of this nomogram model. The poor discriminate ability in predicting the five-year survival probability may be due to the small number of patients over a five-year follow-up in the validation cohort. The predictive ability of this nomogram model for five-year survival probability needs to be further validated in cohorts with more participants and longer follow-ups. As China is a developing country with a lower education level in the elderly, low cognitive level of COPD in patients, and low awareness of symptoms [21], this may lead to better application of this nomogram model in patients in developing countries like China. We also hope for more research to further validate the effectiveness of this nomogram model in population cohorts of different countries in the future. In addition, there are other limitations in the study. This study was limited to one centre, which may lead to reduced representation. However, our subjects included mild to very severe COPD patients, and the study population was collected in eight provinces and over 30 cities in China, which helped to improve the representation of COPD patients. Lastly, the study did not adjust the Charlson comorbidity index due to the limitations of the real-world study, but we have excluded other chronic pulmonary diseases, cancer, severe heart disease, chronic liver disease, and chronic kidney diseases based on personal medical records at baseline which may reduce the confounding deviation of comorbidities to a certain extent.

## CONCLUSIONS

We developed and validated a prognostic nomogram model that accurately predicts mortality across the COPD severity spectrum. The constructed ABEODS nomogram model may have a better prognostic performance than the existing BODEx, updated ADO, and DOSE models in Chinese patients with COPD.

## Additional material


Online Supplementary Document.

## References

[1] Vogelmeier CF Criner GJ Martinez FJ Anzueto A Barnes PJ Bourbeau J Global Strategy for the Diagnosis, Management, and Prevention of Chronic Obstructive Lung Disease 2017 Report. GOLD Executive Summary. Am J Respir Crit Care Med. 2017;195:557–82. 10.1164/rccm.201701-0218PP 28128970

[2] GBD 2019 Diseases and Injuries Collaborators Global burden of 369 diseases and injuries in 204 countries and territories, 1990-2019: a systematic analysis for the Global Burden of Disease Study 2019. Lancet. 2020;396:1204–22. 10.1016/S0140-6736(20)30925-9 33069326 PMC7567026

[3] GBD 2015 Disease and Injury Incidence and Prevalence Collaborators Global, regional, and national incidence, prevalence, and years lived with disability for 310 diseases and injuries, 1990-2015: a systematic analysis for the Global Burden of Disease Study 2015. Lancet. 2016;388:1545–602. 10.1016/S0140-6736(16)31678-6 27733282 PMC5055577

[4] Mercado N Ito K Barnes PJ Accelerated ageing of the lung in COPD: new concepts. Thorax. 2015;70:482–9. 10.1136/thoraxjnl-2014-206084 25739910

[5] Montserrat-Capdevila J Godoy P Marsal JR Barbé F Galván L Risk of exacerbation in chronic obstructive pulmonary disease: a primary care retrospective cohort study. BMC Fam Pract. 2015;16:173. 10.1186/s12875-015-0387-6 26642879 PMC4672528

[6] Dijk WD Bemt L Haak-Rongen S Bischoff E Weel C Veen JC Multidimensional prognostic indices for use in COPD patient care. A systematic review. Respir Res. 2011;12:151. 10.1186/1465-9921-12-151 22082049 PMC3228786

[7] Wada H Ikeda A Maruyama K Yamagishi K Barnes PJ Tanigawa T Low BMI and weight loss aggravate COPD mortality in men, findings from a large prospective cohort: the JACC study. Sci Rep. 2021;11:1531. 10.1038/s41598-020-79860-4 33452329 PMC7810869

[8] Anthonisen NR Wright EC Hodgkin JE Prognosis in chronic obstructive pulmonary disease. Am Rev Respir Dis. 1986;133:14–20. 10.1164/arrd.1986.133.1.14 3510578

[9] Casanova C Marin JM Martinez-Gonzalez C de Lucas-Ramos P Mir-Viladrich I Cosio B Differential Effect of Modified Medical Research Council Dyspnea, COPD Assessment Test, and Clinical COPD Questionnaire for Symptoms Evaluation Within the New GOLD Staging and Mortality in COPD. Chest. 2015;148:159–68. 10.1378/chest.14-2449 25612228

[10] Suissa S Dell’Aniello S Ernst P Long-term natural history of chronic obstructive pulmonary disease: severe exacerbations and mortality. Thorax. 2012;67:957–63. 10.1136/thoraxjnl-2011-201518 22684094 PMC3505864

[11] Lutter JI Jörres RA Welte T Watz H Waschki B Alter P Impact of Education on COPD Severity and All-Cause Mortality in Lifetime Never-Smokers and Longtime Ex-Smokers: Results of the COSYCONET Cohort. Int J Chron Obstruct Pulmon Dis. 2020;15:2787–98. 10.2147/COPD.S273839 33177816 PMC7652228

[12] Song Q Liu C Cheng W Lin L Li T Li X Clinical characteristics and risk of all-cause mortality in low education patients with chronic obstructive pulmonary disease in the Chinese population. J Glob Health. 2023;13:04163. 10.7189/jogh.13.04163 38033249 PMC10693353

[13] Celli BR Predictors of mortality in COPD. Respir Med. 2010;104:773–9. 10.1016/j.rmed.2009.12.017 20417082

[14] Bellou V Belbasis L Konstantinidis AK Tzoulaki I Evangelou E Prognostic models for outcome prediction in patients with chronic obstructive pulmonary disease: systematic review and critical appraisal. BMJ. 2019;367:l5358. 10.1136/bmj.l5358 31585960 PMC6776831

[15] Celli BR Cote CG Marin JM Casanova C Montes de Oca M Mendez RA The body-mass index, airflow obstruction, dyspnea, and exercise capacity index in chronic obstructive pulmonary disease. N Engl J Med. 2004;350:1005–12. 10.1056/NEJMoa021322 14999112

[16] Soler-Cataluña JJ Martínez-García MA Sánchez LS Tordera MP Sánchez PR Severe exacerbations and BODE index: two independent risk factors for death in male COPD patients. Respir Med. 2009;103:692–9. 10.1016/j.rmed.2008.12.005 19131231

[17] Puhan MA Hansel NN Sobradillo P Enright P Lange P Hickson D Large-scale international validation of the ADO index in subjects with COPD: an individual subject data analysis of 10 cohorts. BMJ Open. 2012;2:e002152. 10.1136/bmjopen-2012-002152 23242246 PMC3533065

[18] Jones RC Donaldson GC Chavannes NH Kida K Dickson-Spillmann M Harding S Derivation and validation of a composite index of severity in chronic obstructive pulmonary disease: the DOSE Index. Am J Respir Crit Care Med. 2009;180:1189–95. 10.1164/rccm.200902-0271OC 19797160

[19] Sundh J Janson C Lisspers K Ställberg B Montgomery S The Dyspnoea, Obstruction, Smoking, Exacerbation (DOSE) index is predictive of mortality in COPD. Prim Care Respir J. 2012;21:295–301. 10.4104/pcrj.2012.00054 22786813 PMC6547953

[20] Wang S Yang L Ci B Maclean M Gerber DE Xiao G Development and Validation of a Nomogram Prognostic Model for SCLC Patients. J Thorac Oncol. 2018;13:1338–48. 10.1016/j.jtho.2018.05.037 29902534 PMC7678404

[21] Fan J Wang N Fang LW Feng YJ Cong S Bao HL [Awareness of knowledge about chronic obstructive pulmonary disease and related factors in residents aged 40 years and older in China, 2014]. Zhonghua Liu Xing Bing Xue Za Zhi. 2018;39:586–92. Chinese. 29860799 10.3760/cma.j.issn.0254-6450.2018.05.009

[22] Fang X Wang X Bai C COPD in China: the burden and importance of proper management. Chest. 2011;139:920–9. 10.1378/chest.10-1393 21467059 PMC7125604

[23] Duan JX Cheng W Zeng YQ Chen Y Cai S Li X Characteristics of Patients with Chronic Obstructive Pulmonary Disease Exposed to Different Environmental Risk Factors: A Large Cross-Sectional Study. Int J Chron Obstruct Pulmon Dis. 2020;15:2857–67. 10.2147/COPD.S267114 33192059 PMC7654530

[24] Cheng W Zhou A Zeng Y Lin L Song Q Liu C Prediction of Hospitalization and Mortality in Patients with Chronic Obstructive Pulmonary Disease with the New Global Initiative for Chronic Obstructive Lung Disease 2023 Group Classification: A Prospective Cohort and a Retrospective Analysis. Int J Chron Obstruct Pulmon Dis. 2023;18:2341–52. 10.2147/COPD.S429104 37908629 PMC10615105

[25] Liu C Cheng W Zeng Y Zhou Z Zhao Y Duan J Different Characteristics of Ex-Smokers and Current Smokers with COPD: A Cross-Sectional Study in China. Int J Chron Obstruct Pulmon Dis. 2020;15:1613–9. 10.2147/COPD.S255028 32753861 PMC7354950

[26] Wheaton AG Liu Y Croft JB VanFrank B Croxton TL Punturieri A Chronic Obstructive Pulmonary Disease and Smoking Status - United States, 2017. MMWR Morb Mortal Wkly Rep. 2019;68:533–8. 10.15585/mmwr.mm6824a1 31220055 PMC6586372

[27] Ferrer M Alonso J Morera J Marrades RM Khalaf A Aguar MC Chronic obstructive pulmonary disease stage and health-related quality of life. The Quality of Life of Chronic Obstructive Pulmonary Disease Study Group. Ann Intern Med. 1997;127:1072–9. 10.7326/0003-4819-127-12-199712150-00003 9412309

[28] Hanley JA McNeil BJ A method of comparing the areas under receiver operating characteristic curves derived from the same cases. Radiology. 1983;148:839–43. 10.1148/radiology.148.3.6878708 6878708

[29] Xu W Collet JP Shapiro S Lin Y Yang T Wang C Negative impacts of unreported COPD exacerbations on health-related quality of life at 1 year. Eur Respir J. 2010;35:1022–30. 10.1183/09031936.00079409 19897555

[30] Dugravot A Fayosse A Dumurgier J Bouillon K Rayana TB Schnitzler A Social inequalities in multimorbidity, frailty, disability, and transitions to mortality: a 24-year follow-up of the Whitehall II cohort study. Lancet Public Health. 2020;5:e42–50. 10.1016/S2468-2667(19)30226-9 31837974 PMC7098476

[31] Uchmanowicz B Chudiak A Uchmanowicz I Rosińczuk J Froelicher ES Factors influencing adherence to treatment in older adults with hypertension. Clin Interv Aging. 2018;13:2425–41. 10.2147/CIA.S182881 30568434 PMC6276633

[32] Koehorst-Ter Huurne K Groothuis-Oudshoorn CG vanderValk PD, Movig KL, van der Palen J, Brusse-Keizer M. Association between poor therapy adherence to inhaled corticosteroids and tiotropium and morbidity and mortality in patients with COPD. Int J Chron Obstruct Pulmon Dis. 2018;13:1683–90. 10.2147/COPD.S161374 29872286 PMC5973470

[33] Munari AB Gulart AA Araújo J Zanotto J Sagrillo LM Karloh M Modified Medical Research Council and COPD Assessment Test Cutoff Points. Respir Care. 2021;66:1876–84. 10.4187/respcare.08889 34670858

[34] Tønnesen P Smoking cessation and COPD. Eur Respir Rev. 2013;22:37–43. 10.1183/09059180.00007212 23457163 PMC9487432

[35] Moll M Qiao D Regan EA Hunninghake GM Make BJ Tal-Singer R Machine Learning and Prediction of All-Cause Mortality in COPD. Chest. 2020;158:952–64. 10.1016/j.chest.2020.02.079 32353417 PMC7478228

[36] Oh AS Baraghoshi D Lynch DA Ash SY Crapo JD Humphries SM Emphysema Progression at CT by Deep Learning Predicts Functional Impairment and Mortality: Results from the COPDGene Study. Radiology. 2022;304:672–9. 10.1148/radiol.213054 35579519 PMC9434819

[37] Yun J Cho YH Lee SM Hwang J Lee JS Oh YM Deep radiomics-based survival prediction in patients with chronic obstructive pulmonary disease. Sci Rep. 2021;11:15144. 10.1038/s41598-021-94535-4 34312450 PMC8313653

[38] González G Ash SY Vegas-Sánchez-Ferrero G Onieva Onieva J Rahaghi FN Ross JC Disease Staging and Prognosis in Smokers Using Deep Learning in Chest Computed Tomography. Am J Respir Crit Care Med. 2018;197:193–203. 10.1164/rccm.201705-0860OC 28892454 PMC5768902

