# Supplementary material for: Development and validation of a nomogram model for mortality prediction in stable chronic obstructive pulmonary disease patients: A prospective observational study in the RealDTC cohort
Source: J Glob Health. 2024 Feb 23;14:04049. doi: 10.7189/jogh.14.04049 (PMC10905054; doi:10.7189/jogh.14.04049)
Supplement: Online Supplementary Document. [file jogh-14-04049-s001.pdf]

Table S1. Comparison of demographic and clinical characteristics between training cohort and validation cohort in COPD patients

**Variables**	**Total (n = 2499)**	**Validation cohort (n = 756)**	**Training cohort (n = 1743)**	**P.value**
Male, n (%)	2175 (87.0)	646 (85.4)	1529 (87.7)	0.12
Age, Mean ± SD	63.2 ± 8.2	63.5 ± 8.5	63.1 ± 8.0	0.23
Educational level, n (%)				0.45
Primary/below	1060 (42.4)	326 (43.1)	734 (42.1)	
Middle	875 (35.0)	274 (36.3)	601 (34.5)	
High	407 (16.3)	115 (15.2)	292 (16.7)	
College/upper	157 (6.3)	41 (5.4)	116 (6.7)	
BMI, Mean ± SD	22.6 ± 3.5	22.6 ± 3.5	22.6 ± 3.5	0.87
Smoking status, n (%)				0.32
Non-smoker	418 (16.7)	135 (17.9)	283 (16.2)	
Smoker	2081 (83.3)	621 (82.1)	1460 (83.8)	
FEV_1/FVC%, Median (IQR)	46.0 (37.0, 57.0)	46.0 (37.3, 57.0)	45.9 (36.9, 57.0)	0.98
FEV_1, Median (IQR)	1.22 (0.89,1.67)	1.20 (0.87,1.69)	1.22 (0.90,1.67)	0.77
FEV_1%pred, Mean ± SD	53.1 ± 20.8	53.5 ± 20.5	53.0 ± 21.0	0.56
COPD severity, n (%)				0.08
Mild	266 (10.6)	73 (9.7)	193 (11.1)	
Moderate	1035 (41.4)	327 (43.2)	708 (40.6)	
Severe	856 (34.3)	270 (35.7)	586 (33.6)	
Very severe	342 (13.7)	86 (11.4)	256 (14.7)	
CAT, Mean ± SD	15.5 ± 6.6	15.8 ± 6.6	15.4 ± 6.6	0.24
mMRC, Mean ± SD	2.1 ± 1.0	2.1 ± 1.0	2.1 ± 1.0	0.40
CCQ score, Mean ± SD	2.2 ± 0.7	2.2 ± 0.7	2.2 ± 0.7	0.93
AE in past year, n (%)	1388 (55.5)	426 (56.3)	962 (55.2)	0.59
Severe AE in past year, n (%)	818 (32.7)	250 (33.1)	568 (32.6)	0.81
GOLD group, n (%)				0.71
A	210 (8.4)	67 (8.9)	143 (8.2)	
B	1119 (44.8)	326 (43.1)	793 (45.5)	
C	72 (2.9)	21 (2.8)	51 (2.9)	
D	1098 (43.9)	342 (45.2)	756 (43.4)	
Inhalation therapy, n (%)				0.16
LAMA	812 (32.4)	267 (35.3)	545 (31.3)	
ICS+LABA	234 (9.4)	62 (8.2)	172 (9.9)	
ICS+LABA+LAMA	1299 (52.0)	385 (50.9)	914 (52.4)	
Others	154 (6.2)	42 (5.6)	112 (6.4)	
ICS therapy, n (%)	1535 (61.4)	448 (59.3)	1087 (62.4)	0.14
BODEx index, Mean ± SD	4.0 ± 2.1	4.0 ± 2.1	4.0 ± 2.1	0.64

Updated ADO index, Mean ± SD	7.1 ± 2.4	7.1 ± 2.5	7.0 ± 2.3	0.59
DOSE index, Mean ± SD	3.1 ± 1.6	3.1 ± 1.6	3.1 ± 1.6	0.73
AE in first year, n (%)	853 (34.1)	268 (35.4)	585 (33.6)	0.36
Severe AE in first year, n (%)	571 (22.8)	184 (24.3)	387 (22.2)	0.24
Survival status, n (%)				0.30
Survived	2324 (93.0)	697 (92.2)	1627 (93.3)	
Deceased	175 (7.0)	59 (7.8)	116 (6.7)	
Days followed up, Mean ± SD	39.9 ± 8.2	39.9 ± 8.1	39.8 ± 8.2	0.75

Abbreviations: COPD, chronic obstructive pulmonary disease; BMI, body mass index; FEV_1, forced expiratory volume in one second; FVC, forced vital capacity; IQR, Interquartile range; CAT, COPD assessment test; mMRC, modified medical research council dyspnea Scale; CCQ, clinical COPD questionnaire; AE, acute exacerbation; COPD severity and ABCD group were classified using Global Initiative for Chronic Obstructive Lung Disease (GOLD) criteria; LABA, long-acting β2-agonist; LAMA, long-acting muscarinic antagonist; ICS, inhaled corticosteroid; BODEx, a combination of BMI, airflow obstruction, dyspnea, and exacerbations; ADO, a combination of age, dyspnea, obstruction; DOSE, a combination of the dyspnea, obstruction, smoking, exacerbation.

Table S2. Demographics and clinical characteristics of the COPD patients in the training cohort, according to whether they survived

**Variables**	**Total (n = 1743)**	**Survived (n = 1627)**	**Deceased (n = 116)**	**P.value**
Male, n (%)	1529 (87.7)	1424 (87.5)	105 (90.5)	0.34
Age, Mean ± SD	63.1 ± 8.0	62.8 ± 8.1	67.3 ± 6.4	**< 0.01**
Educational level, n (%)				**< 0.01**
Primary/below	734 (42.1)	664 (40.8)	70 (60.3)	
Middle	601 (34.5)	571 (35.1)	30 (25.9)	
High	292 (16.7)	278 (17.1)	14 (12.1)	
College/upper	116 (6.7)	114 (7.0)	2 (1.7)	
BMI, Mean ± SD	22.6 ± 3.5	22.7 ± 3.4	21.0 ± 3.4	**< 0.01**
Baseline Smoking status, n (%)				0.12
Non-smoker	283 (16.2)	270 (16.6)	13 (11.2)	
Current smoker	928 (53.3)	869 (53.4)	59 (50.9)	
Former smoker	532 (30.5)	488 (30.0)	44 (37.9)	
FEV_1/FVC%, Median (IQR)	45.9 (36.9,57.0)	46.0 (37.0,57.1)	42.3 (33.2,55.8)	**0.01**
FEV_1, Median (IQR)	1.2 (0.9,1.7)	1.2 (0.9,1.7)	1.0 (0.7,1.3)	**< 0.01**
FEV_1%pred, Mean ± SD	53.0 ± 21.0	53.4 ± 20.7	46.6 ± 23.3	**< 0.01**
COPD severity, n (%)				**< 0.01**
Mild	193 (11.1)	186 (11.4)	7 (6.0)	
Moderate	708 (40.6)	674 (41.4)	34 (29.3)	
Severe	586 (33.6)	540 (33.2)	46 (39.7)	
Very severe	256 (14.7)	227 (14.0)	29 (25.0)	
CAT, Mean ± SD	15.4 ± 6.6	15.2 ± 6.5	18.8 ± 7.2	**< 0.01**
mMRC, Mean ± SD	2.1 ± 1.0	2.0 ± 1.0	2.6 ± 0.9	**< 0.01**
CCQ score, Mean ± SD	2.2 ± 0.7	2.1 ± 0.7	2.4 ± 0.7	**< 0.01**
AE in past year, n (%)	962 (55.2)	889 (54.6)	73 (62.9)	0.08
Severe AE in past year, n (%)	568 (32.6)	524 (32.2)	44 (37.9)	0.20
GOLD group, n (%)				**0.01**
A	143 (8.2)	142 (8.7)	1 (0.9)	
B	793 (45.5)	745 (45.8)	48 (41.4)	
C	51 (2.9)	49 (3.0)	2 (1.7)	
D	756 (43.4)	691 (42.5)	65 (56.0)	
Inhalation therapy, n (%)				0.25
LAMA	545 (31.3)	514 (31.6)	31 (26.7)	
ICS+LABA	172 (9.9)	163 (10.0)	9 (7.8)	
ICS+LABA+LAMA	914 (52.4)	843 (51.8)	71 (61.2)	
Others	112 (6.4)	107 (6.6)	5 (4.3)	
ICS therapy, n (%)	1087 (62.4)	1006 (61.8)	81 (69.8)	0.09
BODEx index, Mean ± SD	4.0 ± 2.1	4.0 ± 2.1	4.9 ± 1.9	**< 0.01**
Updated ADO index, Mean ± SD	7.0 ± 2.3	6.9 ± 2.3	8.7 ± 1.9	**< 0.01**

DOSE index, Mean ± SD	3.1 ± 1.6	3.0 ± 1.6	4.1 ± 1.8	**< 0.01**
AE in first year, n (%)	585 (33.6)	507 (31.2)	78 (67.2)	**< 0.01**
Sever AE in first year, n (%)	387 (22.2)	315 (19.4)	72 (62.1)	**< 0.01**
Days followed up, Mean ± SD	39.8 ± 8.2	41.1 ± 6.6	22.3 ± 9.3	**< 0.01**

Abbreviations: COPD, chronic obstructive pulmonary disease; BMI, body mass index; SD, Standard Deviation; FEV_1, forced expiratory volume in one second; FVC, forced vital capacity; IQR, Interquartile range; CAT, COPD assessment test; mMRC, modified medical research council dyspnea Scale; CCQ, clinical COPD questionnaire; AE, acute exacerbation; COPD severity and ABCD group were classified using Global Initiative for Chronic Obstructive Lung Disease (GOLD) criteria; LABA, long-acting β2-agonist; LAMA, long-acting muscarinic antagonist; ICS, inhaled corticosteroid; BODEx, a combination of BMI, airflow obstruction, dyspnea, and exacerbations; ADO, a combination of age, dyspnea, obstruction; DOSE, a combination of the dyspnea, obstruction, smoking, exacerbation.

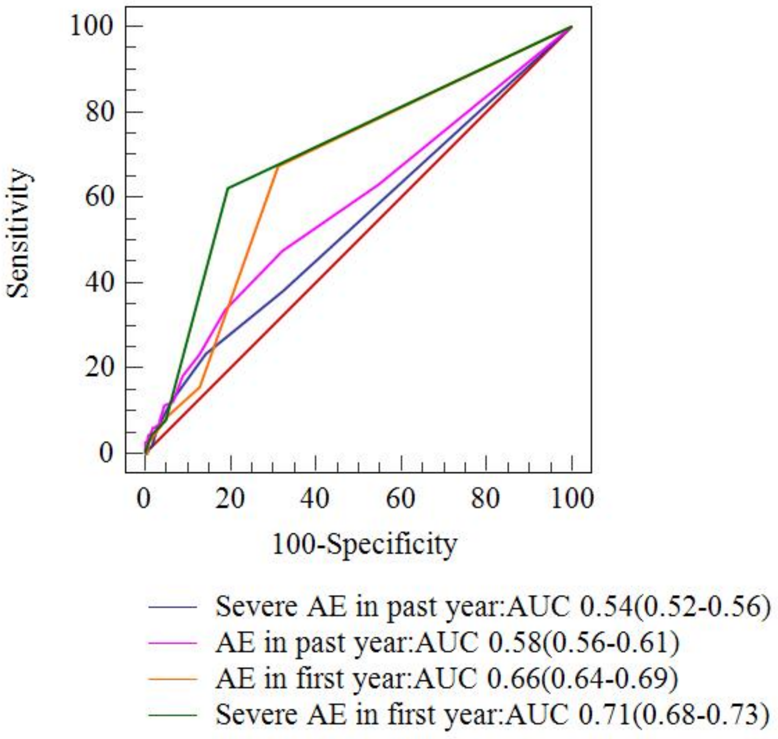

Figure S1. ROC curve analysis between different AE frequencies in the past-year and first-year follow-up.

Abbreviations: ROC, receiver operating characteristic; AUC, the area under the ROC curve; AE, acute exacerbation.

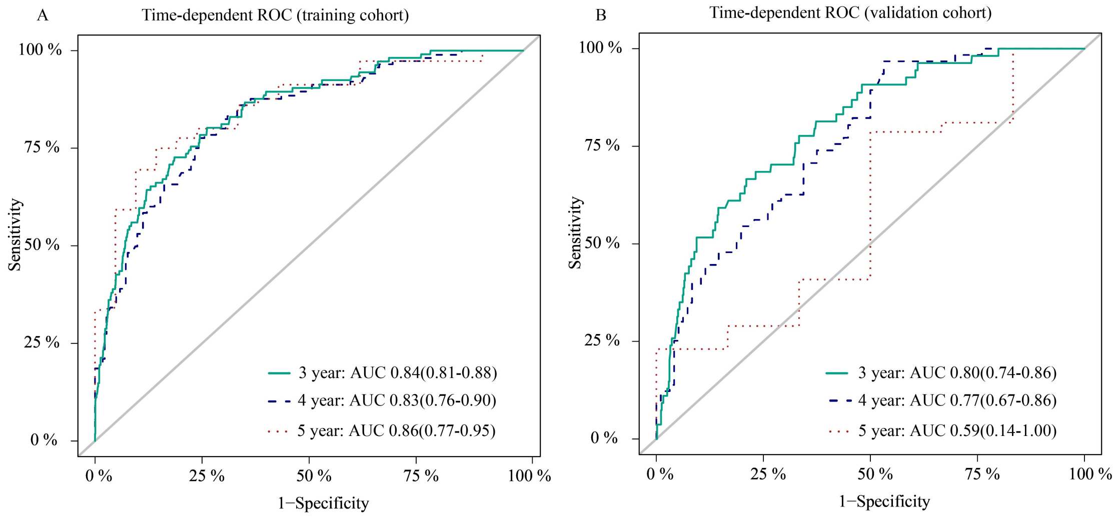

Figure S2. Time-dependent ROC curves of ABEODS nomogram model in the training cohort and validation cohort.

Notes: (A-B) Time-dependent AUC of using the nomogram to predict overall survival probability within 3-year/4-year/5-year in the training cohort (A) and validation cohort (B). The line represents AUC＞0.7, which is considered ideal.

Abbreviations: ROC, receiver operating characteristic; AUC, area under receiver operating characteristic curve; COPD, chronic obstructive pulmonary disease; OS, overall survival; BMI, body mass index; ABEODS, a combination of age, BMI, educational level, airflow obstruction, dyspnea and severe exacerbation in the first-year.

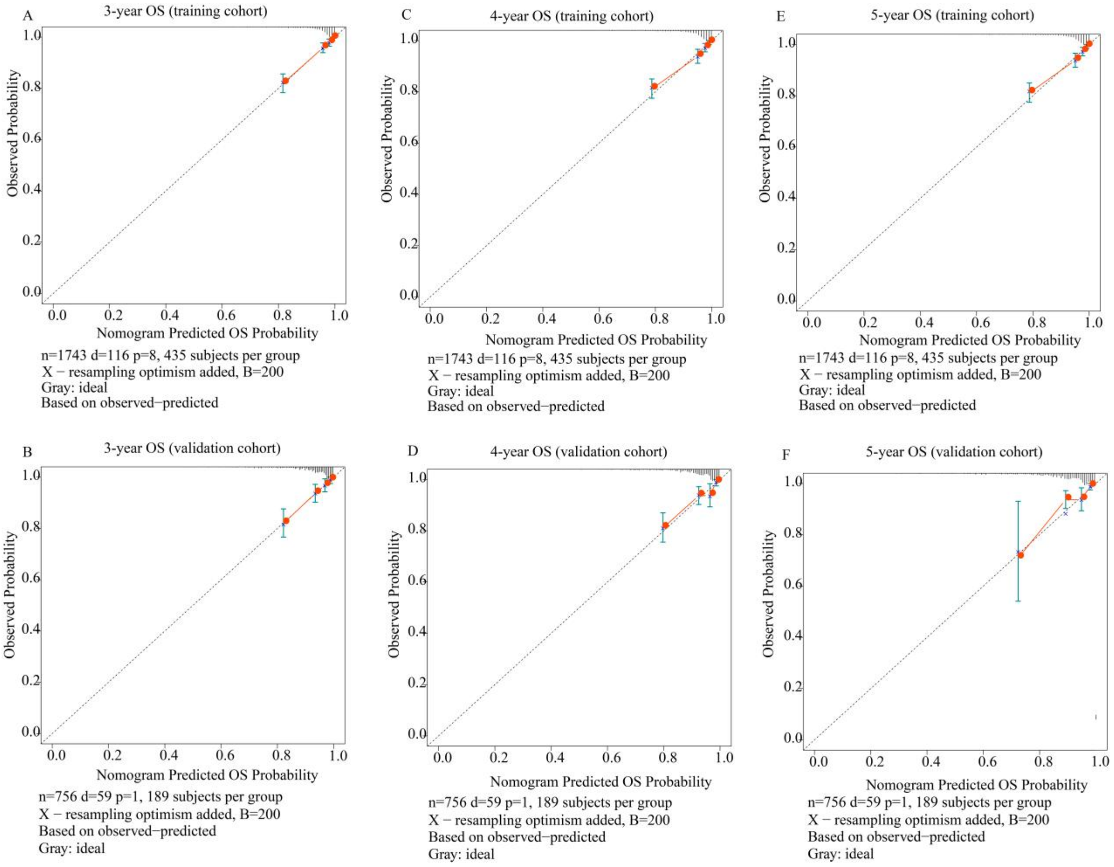

Figure S3. Calibration curves of ABEODS nomogram model in the training cohort and validation cohort.

Notes: (A-B) Calibration curves of 3-year OS probability for COPD patients. (C-D) Calibration curves of 4-year OS probability for COPD patients. (E-F) Calibration curves of 5-year OS probability for COPD patients.

Abbreviations: COPD, chronic obstructive pulmonary disease; OS, overall survival; BMI, body mass index; ABEODS, a combination of age, BMI, educational level, airflow obstruction, dyspnea, and severe exacerbation in the first-year.

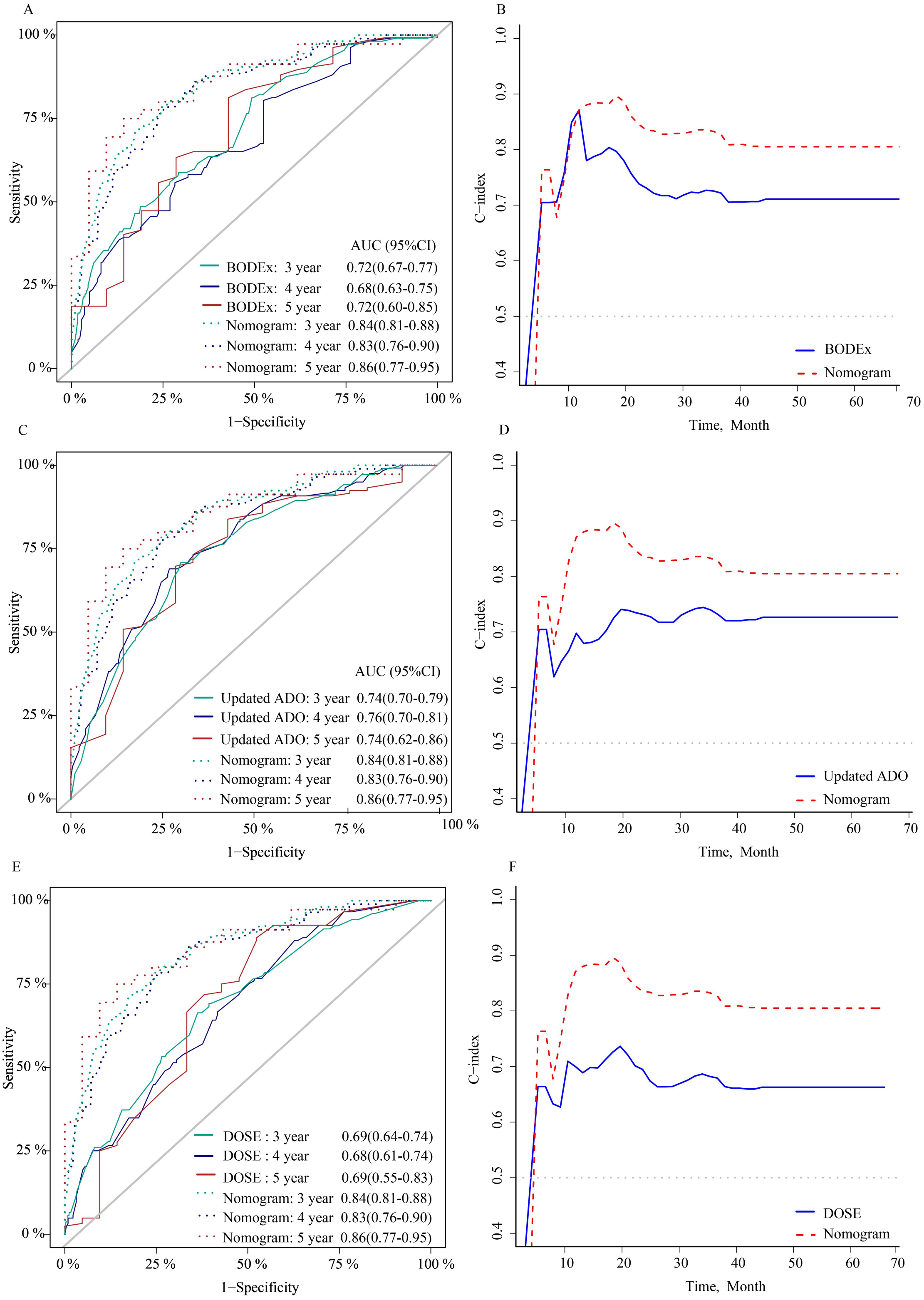

Figure S4. ROC curve and C-index analysis between ABEODS nomogram model and three earlier models.

Notes: (A-B) AUC and C-index between the ABEODS nomogram mortality prediction model and BODEx. (C-D) AUC and C-index between the ABEODS

nomogram mortality prediction model and updated ADO. (E-F) AUC and C-index between the ABEODS nomogram mortality prediction model and DOSE.

Abbreviations: ROC, receiver operating characteristic; AUC, the area under the ROC curve; C-index, concordance index; BMI, body mass index; ABEODS, a combination of age, BMI, educational level, airflow obstruction, dyspnea and severe exacerbation in the first-year; BODEx, a combination of BMI, airflow obstruction, dyspnea, and severe exacerbations frequency in the past-year; ADO, a combination of age, dyspnea and airflow obstruction; DOSE, a combination of dyspnea, airflow obstruction, current smoking status and exacerbation frequency in the past-year.

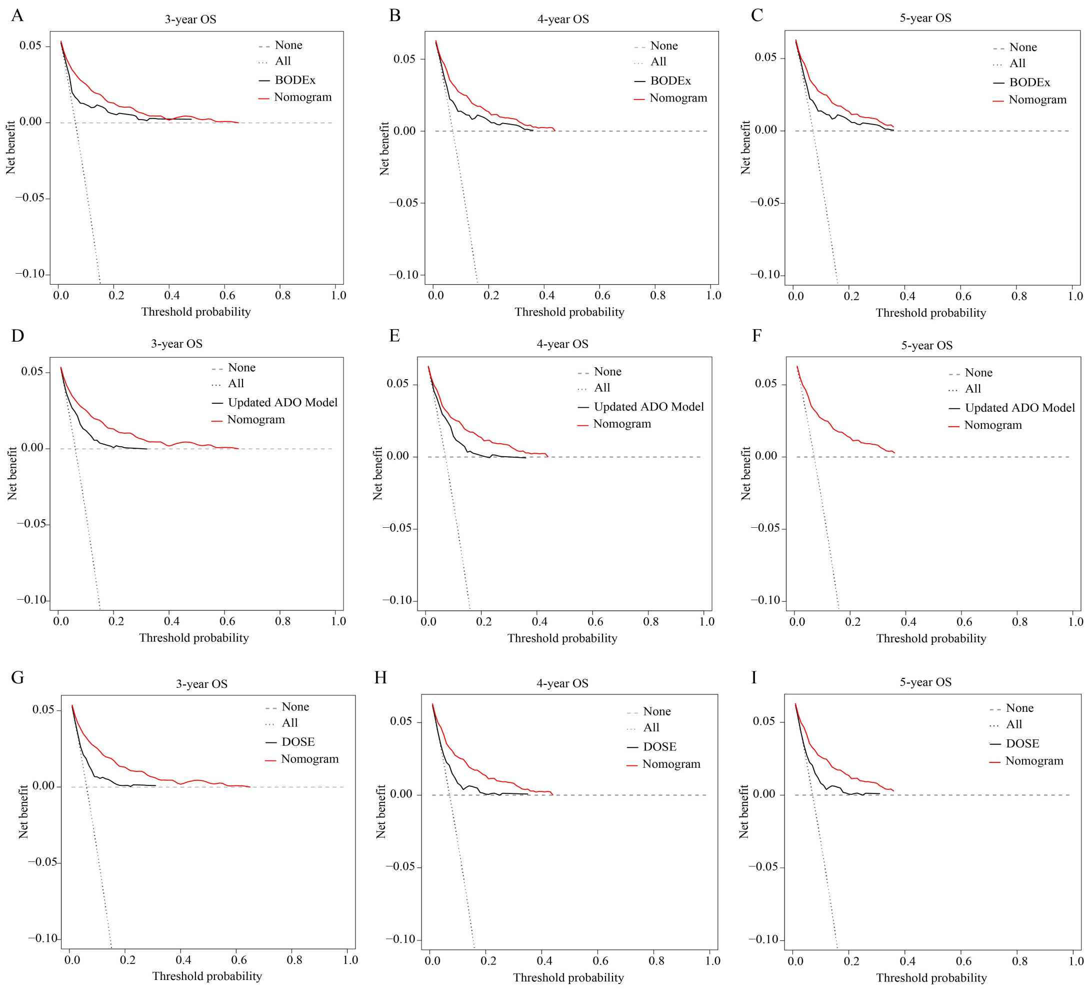

Figure S5. Decision curve analysis between the ABEODS nomogram model and three earlier models.

Notes: (A-C) 3-year/4-year/5-year survival benefit between the ABEODS nomogram mortality prediction model with BODEx. (D-F) 3-year/4-year/5-year survival benefit between the ABEODS nomogram mortality prediction model with updated ADO. (G-I) 3-year/4-year/5-year survival benefit between the ABEODS nomogram mortality prediction model and DOSE.

Abbreviations: ROC, receiver operating characteristic; AUC, the area under the ROC curve; BMI, body mass index; ABEODS, a combination of age, BMI, educational level, airflow obstruction, dyspnea, and severe exacerbation in the first-year; BODEx, a combination of BMI, airflow obstruction, dyspnea, and severe exacerbations frequency in the past-year; ADO, a combination of age, dyspnea and airflow

obstruction; DOSE, a combination of dyspnea, airflow obstruction, current smoking status and exacerbation frequency in the past-year.

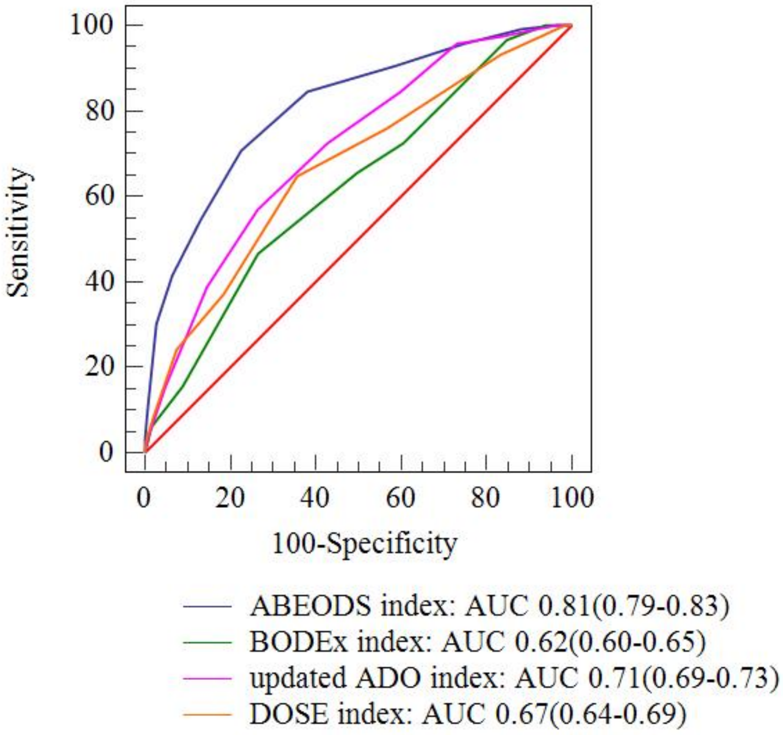

Figure S6. ROC curve analysis between ABEODS index, BODEx index, updated ADO index, and DOSE index.

Abbreviations: ROC, receiver operating characteristic; AUC, the area under the ROC curve; BMI, body mass index; ABEODS, a combination of age, BMI, educational level, airflow obstruction, dyspnea, and severe exacerbation in the first-year; BODEx, a combination of BMI, airflow obstruction, dyspnea, and severe exacerbations frequency in the past-year; ADO, a combination of age, dyspnea and airflow obstruction; DOSE, a combination of dyspnea, airflow obstruction, current smoking status and exacerbation frequency in the past-year.
